# A Word as to Papoid

**Published:** 1887-09

**Authors:** Willard H. Morse

**Affiliations:** Westfield, N. J.


### *A WORD AS TO PAPOID.*

By WILLARD H. MORSE, M. D., Westfield, N. J.

Among some forty new remedies which I have studied therapeutically and chemically, with a view of discussing them in a work supplemental to my book on "New Therapeutical Agents," there is no one that has interested me more or commanded such especial faith as papoid, the well-named vegetable pepsin, a powder prepared from the juice of the fruit of the *carica papaia*, a South American tree, and introduced into this country quite recently. While I do not doubt that many medical men who have employed it will agree with me in saying a "good word" for it, there may be others who are holding it *sub judice*, and may find it an encouragement to its adoption to know that it is most heartily endorsed by such physicians as Professors Finckler, of Bonn; Rossbach, of Jena; Kuhne, of Heidelberg, and other Germans (as Oertel, Albrecht, Tussca, Bouchut, Kohts, Asch, Chaeffer, Heinemann, Croner, Leyden, Drier, Fraentzel and Kriege); in England by Griffith Hughes, George Herschell, Langley and others; and in this country by Abraham Jacobi, Andrew H. Smith, Frederick M. Dearborne and many others. For myself, I could not ask for an endorsement from a higher or more satisfactory authority than the President of the New York Academy of Medicine (Dr. Jacobi).

Leaving out my own experience, these several physicians agree in saying of papoid—

1. That its ability for conversion of albumen into peptones is five times greater than that of pepsin.

2. It stimulates the peptic glands.

3. It is antiseptic and hypnotic.

4. It has a duodenal action.

5. It relieves dyspepsia, diphtheria, anorexia, gastritis, gastric catarrh, and neuralgia of the stomach.

6. The dose is, grs. i–v.

7. It is more than a substitute for pepsin, as in addition to doing all that pepsin does, it acts in presence of an alkali or an acid.

8. It dissolves fibrin, and necessarily the diphtheritic membrane when used as a solution or as a spray.

If more is needed to bring the drug to favor, it might be added that the Brazilian Indians soak their meat in this juice to make it tender, and to preserve it, eating the fruit itself for dyspepsia.

